# Underwater Photosynthesis of Submerged Plants – Recent Advances and Methods

**DOI:** 10.3389/fpls.2013.00140

**Published:** 2013-05-21

**Authors:** Ole Pedersen, Timothy D. Colmer, Kaj Sand-Jensen

**Affiliations:** ^1^The Freshwater Biological Laboratory, Department of Biology, University of CopenhagenHillerød, Denmark; ^2^Institute of Advanced Studies, The University of Western AustraliaCrawley, WA, Australia; ^3^School of Plant Biology, The University of Western AustraliaCrawley, WA, Australia

**Keywords:** flooding tolerance, light extinction, carbon dioxide, wetland plants, photorespiration

## Abstract

We describe the general background and the recent advances in research on underwater photosynthesis of leaf segments, whole communities, and plant dominated aquatic ecosystems and present contemporary methods tailor made to quantify photosynthesis and carbon fixation under water. The majority of studies of aquatic photosynthesis have been carried out with detached leaves or thalli and this selectiveness influences the perception of the regulation of aquatic photosynthesis. We thus recommend assessing the influence of inorganic carbon and temperature on natural aquatic communities of variable density in addition to studying detached leaves in the scenarios of rising CO_2_ and temperature. Moreover, a growing number of researchers are interested in tolerance of terrestrial plants during flooding as torrential rains sometimes result in overland floods that inundate terrestrial plants. We propose to undertake studies to elucidate the importance of leaf acclimation of terrestrial plants to facilitate gas exchange and light utilization under water as these acclimations influence underwater photosynthesis as well as internal aeration of plant tissues during submergence.

Knowledge of plant and environmental factors determining photosynthesis by submerged plants is essential for understanding aquatic plant ecophysiology and ecosystem productivity, as well as submergence tolerance of terrestrial plants. Following the pioneering studies by Arens (1933) and Steemann Nielsen (1946) on the use of dissolved inorganic carbon (DIC) for photosynthesis of aquatic plants, numerous studies on the regulatory role of light and DIC for underwater photosynthesis of aquatic plants have been conducted. Particularly, the use of DIC by aquatic plants has fascinated researchers and been reviewed several times (e.g., Madsen and Sand-Jensen, 1991; Maberly and Madsen, 2002; Raven and Hurd, 2012) because this process is important for growth and survival and the uptake mechanisms are very different from those of terrestrial, amphibious, and floating leaved plants exposed to atmospheric air (definitions of these life forms and examples of species are in Sculthorpe (1967)). Since the physical conditions differ markedly between water and air, we have often been approached by researchers asking for practical advice, unavailable in the literature, before engaging in work with underwater photosynthesis. Thus, this review serves to offer the background and a practical guide for measurements of carbon fixation by plants when under water.

Moreover, a growing number of researchers are interested in tolerance of terrestrial plants during flooding (Figure 1A). Torrential rains sometimes result in overland floods that inundate terrestrial plants (Vervuren et al., 2003) and with the current projection on climate change, the frequency of such flooding events are expected to increase (Parry et al., 2007). We therefore predict that research on underwater photosynthesis will extend greatly beyond its current focus on aquatic plants as natural wetlands and many crops will become submerged in future flooding events. Researchers engaging in underwater photosynthesis should be aware that physical restrictions on light availability and gas exchange are much more profound under water than on land (Sand-Jensen and Krause-Jensen, 1997). Moreover, the aquatic sources and mechanisms of inorganic carbon use are complex, difficult to study, and often challenging to fully understand (Madsen and Sand-Jensen, 1991; Raven and Hurd, 2012).

**Figure 1:**
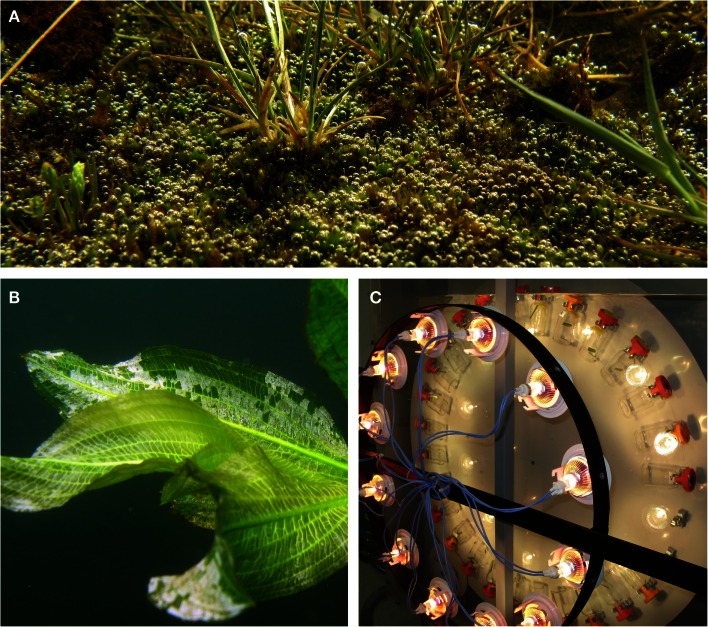
**Completely submerged terrestrial vegetation (A), white flakes of CaCO_3_ on leaves of a pondweed (*Potamogeton lucens*) (B) and an incubator with a vertically rotating wheel holding vials with leaf segments for measurements of underwater net photosynthesis (C) or dark respiration when in complete darkness**. The bubbles on the submerged mosses **(A)** are obvious signs that underwater photosynthesis takes place with O_2_ evolution causing the bubble formation. Moreover, the submerged grasses possess superhydrophobic self cleansing leaf surfaces that retain a thin gas film when under water, evident as a silvery reflecting surface. In **(B)**, high pH on the adaxial leaf surfaces following extensive underwater photosynthesis has resulted in precipitation of CaCO_3_ (See “Challenges Under Water – Reduced Gas Diffusion and Light Penetration”). Photos: a shallow puddle on Öland, Sweden **(A)**, the bicarbonate rich (1.8 mmol DIC L^−1^) Lake Slåen, Denmark **(B)** and the custom built wheel incubator by Ray Scott at the University of Western Australia **(C)**; photos by Ole Pedersen.

Photosynthesis provides sugars and O_2_. The importance of underwater photosynthesis to internal O_2_ status (Figure 1B), including via internal long-distance transport into roots growing in anoxic sediments, has been demonstrated for aquatic species (e.g., Borum et al., 2005; Sand-Jensen et al., 2005; Holmer et al., 2009; Pedersen et al., 2011) and terrestrial wetland plants when completely submerged (Pedersen et al., 2006; Winkel et al., 2011, 2013). By contrast, during the night submerged plants rely on O_2_ uptake from the surrounding water to sustain their respiration and belowground organs can suffer from O_2_ deficiency.

The majority of studies on photosynthesis by submerged aquatic plants have been carried out on detached leaves and algal thalli. These may experience very different environmental conditions than entire communities of submerged plants or plant dominated ecosystems, which have rarely been examined (e.g., Sand-Jensen et al., 2007; Christensen et al., 2013). We thus recommend undertaking studies on communities and ecosystems because they may reveal very different principles of regulation of greater relevance for the ecology and natural performance of submerged aquatic plants, as well as survival of terrestrial species during overland floods.

With the present review, we describe the general background and the recent advances in underwater photosynthesis of phytoelements (shoots, excised thalli, or leaf portions), communities, and plant dominated aquatic ecosystems and present contemporary methods tailor made to quantify photosynthesis and carbon fixation under water.

## Underwater Photosynthesis

### Challenges under water – reduced gas diffusion and light penetration

The 10^4^-fold lower diffusion coefficient of gases in water, compared with in air, presents a major challenge to submerged plants (Armstrong, 1979; Maberly and Madsen, 2002). Diffusive boundary layers (DBL) develop on all surfaces and their thickness adjacent to leaves in water is of the same order of magnitude as those for leaves in air (Vogel, 1994; Raven and Hurd, 2012). Although the transport distance for gases across the DBL is similar, the much lower diffusion coefficient in water results in a 10^4^-fold lower flux for the same concentration gradient and thus the DBL constitutes a much larger proportion of the total resistance to gas exchange for leaves under water than in air (Maberly and Madsen, 2002). The “bottleneck effect” of the DBL on underwater gas exchange was demonstrated in a study of four submerged aquatic species, where the DBL accounted for 90% of the total resistance to carbon fixation (Black et al., 1981). Hence, inorganic carbon limitation of photosynthesis is a much more common and prominent feature for aquatic than terrestrial leaves (Stirling et al., 1997). On average, the underwater photosynthesis increased threefold in a study of 14 submerged freshwater species at saturating relative to ambient supply of DIC supply (Nielsen and Sand-Jensen, 1989). The immediate acclimation of photosynthesis of five fast growing annual terrestrial species by doubling of atmospheric CO_2_ was 1.6- to 2.1-fold while the average increase of relative growth rate over 56 day was 1.25-fold and only significant for one of the five species (Stirling et al., 1998).

**Figure 2:**
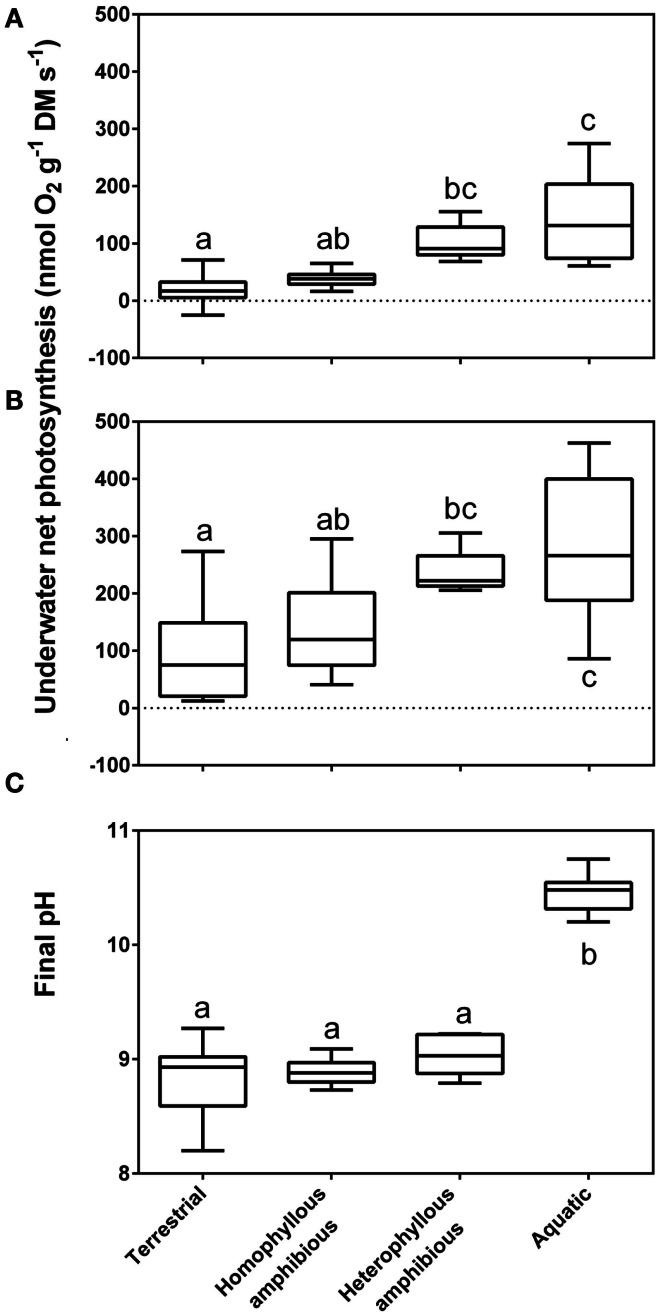
**Rates of underwater net photosynthesis (nmol O_2_ g^−1^ DM s^−1^) in ambient [(A), 90–400 μmol CO_2_ L^−1^] and CO_2_ enriched water [(B), 800 μmol CO_2_ L^−1^] and final pH from a pH drift experiment; Section “pH Drift Approach to Establish CO_2_ Compensation Points” (C) in four groups of life forms defined in Sculthorpe (1967)**. The box whisker plot indicates the range of the observations (bars), 50% of the observations (boxes) and the median (horizontal line). The amount of free CO_2_ present at median final pH of the four groups was 6.20, 6.00, 4.80, and 0.04 μmol L^−1^, respectively. Leaves of aquatic plants species (*n* = 10), heterophyllous amphibious plants (*n* = 5), homophyllous amphibious plants (*n* = 7) were all produced under water while leaves of terrestrial plant species were formed in air (*n* = 12). Letters indicate significant differences (*P* < 0.05); Tukey test. Data from Sand-Jensen et al. (1992), with measurements taken at 15°C.

The restricted gas exchange under water has resulted in evolution of a suite of adaptive features in aquatic leaves and macroalgal thalli to reduce the influence of DBL on the exchange of O_2_ and CO_2_, including the supplementary use of ${\text{HCO}}_{3}^{-}$ (bicarbonate) (Sculthorpe, 1967; Maberly, 1990; Colmer et al., 2011). In seawater and in many freshwaters, the pool of ${\text{HCO}}_{3}^{-}$ is several fold higher than of CO_2_ and thus presents an attractive alternative to CO_2_. Use of ${\text{HCO}}_{3}^{-}$ has evolved many times among unicellular algae, macroalgae, and angiosperms in freshwater and marine environments (Raven and Hurd, 2012) and can involve direct uptake into the cells or external conversion to CO_2_ in the DBL catalyzed by surface bound carbonic anhydrase and/or extrusion of protons in acids bands (charophytes; Lucas and Smith, 1973) or lower leaf surfaces in e.g., species of *Potamogeton* and *Elodea* (Prins et al., 1980) often with precipitation of CaCO_3_ on the alkaline upper leaf surfaces (Figure 1B). While the DBL reduces the direct ${\text{HCO}}_{3}^{-}$ flux to the leaf surface, the “stagnant” layer is required to forming high CO_2_ concentrations by acid titration of ${\text{HCO}}_{3}^{-}$ (Helder, 1985). Use of ${\text{HCO}}_{3}^{-}$ is prominent for marine macroalgae and seagrasses, freshwater charophytes, and in 50% of 80 tested species of freshwater angiosperms (Sand-Jensen and Gordon, 1984; Maberly and Madsen, 2002), but the ability is absent among mosses and pteridophytes. Also 12 amphibious species alternating between emergent and submerged forms in Danish lowland streams relied solely on CO_2_ use although high ${\text{HCO}}_{3}^{-}$ concentrations may still benefit photosynthesis by stabilizing pH and permitting rapid uncatalyzed replenishment of the CO_2_ consumed (Maberly, 1990). The proportion of ${\text{HCO}}_{3}^{-}$ users among angiosperm species in lakes increases significantly with alkalinity and, thus, availability of ${\text{HCO}}_{3}^{-}$ (Maberly and Madsen, 2002) in accordance with the increasing advantage of ${\text{HCO}}_{3}^{-}$ use for photosynthesis and growth. Assuming for simplicity a 10-fold higher apparent preference for CO_2_ than ${\text{HCO}}_{3}^{-}$ by leaves in alkaline water containing 0.015 mmol L^−1^ CO_2_ and 1.5 mmol L^−1^ ${\text{HCO}}_{3}^{-}$, the supply rate of ${\text{HCO}}_{3}^{-}$ would be 10-fold higher than that of CO_2_. In softwater containing only 0.15 mmol L^−1^ ${\text{HCO}}_{3}^{-}$ the supply rate of the two carbon species would be the same. Terrestrial plant species lack these adaptive features for aquatic life, and when underwater their leaves show dramatically reduced net photosynthesis (Sand-Jensen et al., 1992; Nielsen, 1993) and dark respiration (Colmer and Pedersen, 2008; Pedersen et al., 2009). Thus, 13 terrestrial species submerged in CO_2_ rich stream water were unable to use ${\text{HCO}}_{3}^{-}$ and median rates of underwater net photosynthesis were sevenfold lower than of 10 permanently submerged stream plants and the terrestrial species were unable to support substantial growth (Sand-Jensen et al., 1992; Figure 2).

The extraction capacity of the DIC pool is only some 1–4% for obligate CO_2_ users while it is typically 40–70% among ${\text{HCO}}_{3}^{-}$ users; 16 of 19 species (Madsen and Sand-Jensen, 1991). This is because of the ability of ${\text{HCO}}_{3}^{-}$ users to continue photosynthesizing despite very high external pH and low DIC in the water. In vegetation rich water bodies of high pH, ${\text{HCO}}_{3}^{-}$ users can eventually out compete all obligate CO_2_ users (Sand-Jensen et al., 2010). Submerged aquatic plants unable to use ${\text{HCO}}_{3}^{-}$ typically have final pH’s in the external medium of the order of 8.6–9.8 in alkaline solutions and final CO_2_ concentrations equivalent to CO_2_ compensation points of 2–10 μmol L^−1^, while active ${\text{HCO}}_{3}^{-}$ users typically have final pH’s of 9.8–11.0 and final CO_2_ concentrations mostly below 0.3 μmol L^−1^ (Sand-Jensen et al., 1992). For a large collection of stream plants, median final CO_2_ values among the supposedly obligate CO_2_ users were 6.0 μmol L^−1^ for homophyllous and 4.8 μmol L^−1^ for heterophyllous amphibious plants, within the typical range of CO_2_ compensation points, while the median final CO_2_ concentration for the putative ${\text{HCO}}_{3}^{-}$ users was only 0.04 μmol L^−1^ reflecting the supplementary use of ${\text{HCO}}_{3}^{-}$ (Figure 2). Heterophyllous amphibious species form morphologically and anatomically distinct leaf types under water as compared to in air (Sculthorpe, 1967). The underwater leaf forms are an acclimation that enhances underwater gas exchange (Sand-Jensen et al., 1992; Colmer et al., 2011).

Photosynthesis of submerged aquatic plants and flooded terrestrial plants can also be severely limited by light (Kirk, 1994). In water, light is exponentially attenuated with depth following the equation: ${\text{I}}_{Z}={\text{I}}_{0}\left(1-\text{f}\right)\;e^{-Z\varepsilon }$; where I_z_ is the available irradiance at a given depth (*z*), I_0_ is the irradiance at the surface, and ε is the attenuation coefficient. The proportional reflection and back scattering at the water surface (f) is variable but typically about 0.1 such that the proportion of down welling irradiance is 0.9 (Kirk, 1994). The attenuation coefficient of pure water averaged across the photosynthetic spectrum is about 0.03 m^−1^, so in ultra clear water such as oligotrophic oceanic water, rooted plants could grow as deep as 70 m with 10% of surface irradiance still being available, which happens to be the approximate lower depth limit of seagrasses (Duarte, 1991). In most cases, however, colored dissolved organic matter (CDOM), pigments in planktonic algae and suspended particles, together reduce light penetration much more profoundly (Staehr et al., 2012b). Because freshwaters compared with marine waters are typically richer in nutrients, phytoplankton, CDOM exported from land and particles suspended from shallow sediments, attenuation coefficients typically range from 0.3 to 10 m^−1^ and thus have lower depth limits of rooted plants from 7 m to only 0.2 m (Middelboe and Markager, 1997). Flooding after heavy rain is commonly associated with erosion, high particle loads and high release of CDOM from inundated terrestrial soils. Flooded terrestrial plants can, therefore, experience extreme shading corresponding to attenuation coefficients between 1 and 8 m^−1^ (Parolin, 2009) making light limitation also of terrestrial plants a prominent feature during flooding events (Colmer et al., 2011).

### Underwater photosynthesis in submerged aquatic plants and recent advances

The net process of photosynthesis (Eq. 1) is often described simply as the fixation of CO_2_ (or ${\text{HCO}}_{3}^{-}$ in water; Eq. 2) catalyzed by several enzymes, including Rubisco, driven by light and resulting in production of organic matter, O_2_ and OH^−^:

(1) $$\begin{array}{lll} & \text{C}{\text{O}}_{2}+{\text{H}}_{2}\text{O}\to \text{C}{\text{H}}_{2}\text{O}+{\text{O}}_{2} & \end{array}$$

(2) $$\begin{array}{lll} & \text{HC}{{\text{O}}_{3}}^{-}+{\text{H}}_{2}\text{O}\to \text{C}{\text{H}}_{2}\text{O}+{\text{O}}_{2}+\text{O}{\text{H}}^{-} & \end{array}$$

The rate of the process can be determined by the production of O_2_ and new organic matter (e.g., by isotopic tracing with ^13^C and ^14^C) and the consumption of CO_2_, ${\text{HCO}}_{3}^{-}$, or more generally the pool of DIC: CO_2_, ${\text{HCO}}_{3}^{-}$ and ${\text{CO}}_{3}^{2-}$. Photosynthesis is an alkalinization process as reflected by the release of OH^−^ in Eq. 2 and the equivalent consumption of CO_2_ and protons in Eq. 1 (i.e., CO_2_ + H_2_O ↔ H^+^ + ${\text{HCO}}_{3}^{-}$) such that photosynthesis can be determined by the pH increase when converted to DIC consumption accounting for the buffer capacity [mainly due to carbonate alkalinity (CA), See, The CO_2_ Equilibria in Water].

In charophytic macroalgae, use of ${\text{HCO}}_{3}^{-}$ in photosynthesis can be closely coupled stochiometrically to carbonate precipitation (McConnaughey, 1991):

(3) $$\text{C}{\text{a}}^{2+}+2{\text{HCO}}_{3}^{-}+{\text{H}}_{2}\text{O}\to \text{C}{\text{H}}_{2}\text{O}+{\text{O}}_{2}+\text{CaC}{\text{O}}_{\text{3}}$$

This process is pH neutral because conversion of ${\text{HCO}}_{3}^{-}$ to ${\text{CO}}_{3}^{2-}$ generates the necessary proton for conversion of ${\text{HCO}}_{3}^{-}$ to CO_2_ for assimilation. Thus, ${\text{HCO}}_{3}^{-}$ is equally divided between production of organic matter and CaCO_3_ and the photosynthetic quotient (PQ: mol O_2_ evolved mol^−1^ DIC consumed) is only about 0.5 compared with the typical value of 1.0 in Eqs 1 and 2. The active processes are apparently active antiport of H^+^ and Ca^2+^ in the acid bands and Ca^2+^ extrusion in the alkaline bands resulting in gradual accumulation of CaCO_3_ from inside the carbonate layer (McConnaughey, 1991). Carbonate precipitation closely coupled to photosynthesis is also found in coralline red algae, several green algae, and numerous freshwater angiosperms developing polar leaves with high pH and carbonate precipitation being confined to the upper leaf surfaces (Raven and Hurd, 2012). However, it remains to be tested whether active Ca^2+^ extrusion is involved in angiosperm use of ${\text{HCO}}_{3}^{-}$ as in charophytes (Prins et al., 1980). Even though photosynthesis and carbonate precipitation may not be closely coupled, the alkalinization process in Eqs 1 and 2 may still result in carbonate precipitation on leaf surfaces (Figure 1B) or in the surrounding water because of increase of pH and ${\text{CO}}_{3}^{2-}$, though with a variable ratio to the fixation of CO_2_ in organic matter. Consequently, O_2_ evolution is a more reliable measure of underwater photosynthesis, while DIC use and production of organic matter and carbonate are essential parameters in the analysis of plant growth and carbon dynamics in ecosystems and on regional and global scales (McConnaughey et al., 1994; Cole et al., 2007). While coupled calcification photosynthesis leads to extensive drawdown of DIC and sediment accumulation of organic carbon and carbonates, carbonate formation *per se* generates H^+^ tending to reduce pH and increase CO_2_. During geological periods of intense formation of coral reefs, CO_2_ concentrations are suggested to have increased in the ocean and the atmosphere (Opdyke and Walker, 1992). The coupled photosynthesis calcification process has three major ecological or physiological implications: (i) in coral and coralline algae carbonates are directly used to build up the structural skeleton, (ii) in all phototrophs, surface precipitates will protect them against grazing animals, and (iii) calcification will prevent alkalinization during intensive photosynthesis which could otherwise have led to such high pH levels that tissues are damaged and photosynthesis is severely inhibited.

The photosynthetic capacity under optimum light, temperature, and DIC conditions varies among species and within species depending on their investment in active transport processes and catalytic machinery. On dry mass basis, maximum rates of photosynthesis of detached leaves of submerged aquatic plants from lakes typically vary 25-fold and dark respiration only fourfold between slow-growing, oligotrophic isoetid species and fast growing, eutrophic elodeid species (Nielsen and Sand-Jensen, 1989). Photosynthetic rates per unit dry mass increase significantly with reduced leaf thickness, higher relative surface area, and higher concentrations of pigments and nitrogen in structural and catalytic proteins, including Rubisco (Madsen et al., 1993). Because metabolism on a dry mass basis increases with declining leaf thickness, photosynthesis expressed per surface area only varies eightfold among species (Nielsen and Sand-Jensen, 1989). A comparison with terrestrial leaves characterizes the aquatic leaves by their lower chlorophyll and Rubisco concentrations and lower photosynthetic rates per surface area mainly due to the thin leaves of most aquatic species. This finding has been interpreted by Maberly and Madsen (2002) as a result of selection of submerged plants to match the low rates of carbon influx predominantly because of high transport resistance. Thin submerged leaves with chloroplasts in epidermal layers will also increase the cost effectiveness of light use and can also be regarded as a particular advantage in a low light aquatic environment with no risk of desiccation damage to the epidermal layers.

**Figure 3:**
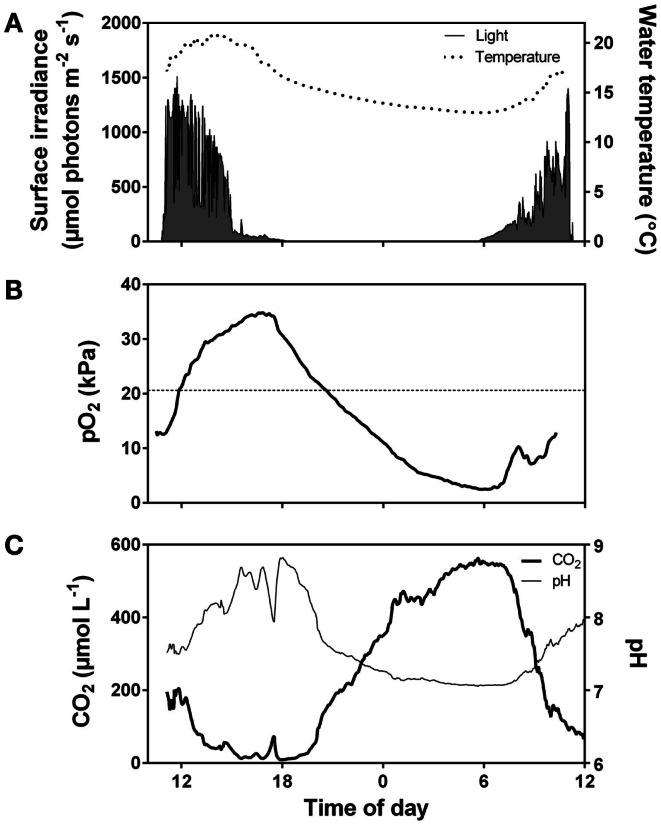
**Diurnal fluctuations in surface irradiance and water temperature (A), O_2_ partial pressure (B), and CO_2_ concentration and pH (C) in the water of a vegetation rich pond in Western Australia**. The pond was densely vegetated by *Meionectes brownii* which in late afternoon had extracted CO_2_ from the water down to below air equilibrium (<15 μmol L^−1^). During the night, total system respiration consumed O_2_ (pO_2_ dropped to 2.5 kPa early in the morning) and CO_2_ rose to 550 μmol L^−1^. Data extracted from Rich et al. (2013).

Realized rates of underwater photosynthesis for a given plant tissue varies from zero at compensation levels for light and DIC to maximum rates at saturating light and DIC. Light and DIC levels required to saturate photosynthesis increase with temperature and are highly dependent on the extent of self shading and, therefore, the scale of analysis of either detached leaves, individuals or populations (See Underwater Photosynthesis – Approaches and Methods). On a daily level, light limitation takes place early in the morning (low light but plenty of CO_2_, Figure 3) and co-limitation of both light and CO_2_ takes place late in the afternoon where also CO_2_ is low (Figure 3). On a seasonal level, light limitation is present from late autumn to early spring outside the tropical regions. Populations in deep or turbid waters and dense populations with high self shading face permanent light limitation. Photosynthetic rates at saturating light and DIC increase with temperature due to stimulation of enzyme activity up to an optimum level depending on the adaptation and acclimation of the species but are usually located between 25 and 32°C for temperate submerged aquatic plants (Santamaría and van Vierssen, 1997). The temperature exponent gradually declines and reaches zero as temperature approaches the optimum temperature and it turns negative above the optimum due to increasing influence of photorespiration (Long, 1991) and risk of damage of macromolecules, membranes, and structural organization of membrane proteins (Johnson et al., 1974; Lambers et al., 2008). Respiration continues to increase up to higher temperatures than photosynthesis resulting in proportionally greater losses of organic matter and optima for growth being located at lower temperature than optima for photosynthesis (Olesen and Madsen, 2000; Pilon and Santamaría, 2001).

Ninety nine percent of all studies of aquatic photosynthesis have been carried out with detached leaves or thalli and this selectiveness influences the perception of the regulation of aquatic photosynthesis (Sand-Jensen and Krause-Jensen, 1997). The influence of light, DIC, and temperature on underwater photosynthesis show mutual interdependencies and are, moreover, strongly dependent on the spatial scale. From detached phytoelements to closed communities, light compensation points typically increase three- to eightfold and light saturation levels increase from 200 to 400 μmol m^−2^ s^−1^ to more than the maximum irradiances at noon of about 1500 μmol m^−2^ s^−1^ (Table 1; Sand-Jensen et al., 2007). The stimulation of photosynthesis in alkaline water by rising CO_2_ concentrations from 20 (close to air equilibrium) to 250 μmol L^−1^ (more than 10-fold above air equilibrium) is about ninefold for detached leaves and only 1.9- to 2.5-fold for dense communities of freshwater CO_2_ users while for efficient ${\text{HCO}}_{3}^{-}$ users the CO_2_ stimulation is only about twofold for individual leaves and insignificant for dense communities (Table 2; Sand-Jensen et al., 2007). Open communities of less self shading take an immediate position between detached individual leaves and dense communities. Profound self shading and light limitation of photosynthesis in dense aquatic communities imply that the influence of temperature and inorganic carbon supply is smaller than observed for well illuminated phytoelements. The full scale influence of temperature and CO_2_ on community photosynthesis is confined to tissues in the upper part of the canopy receiving irradiances above light saturation.

**Table 1:** **Photosynthetic parameters for thallus segments and communities of *Fucus serratus* of varying leaf area index (LAI)**.

	Thallus (mean)	Community LAI (m^2^ m^−2^)			
		3.0	6.3	8.9	13.8
GP_max_ (μmol O_2_ m^−2^ s^−1^)	7.95	11.5	17.4	22.0	23.7
A (μmol O_2_ mol^−1^ photon)	0.064	0.031	0.049	0.069	0.072
E_C_ (μmol photon m^−2^ s^−1^)	22	67	99	94	175
E_K_ (μmol photon m^−2^ s^−1^)	Approx. 300	Not sat.	Not sat.	Not sat.	Not sat.

*GP_max_: light saturated photosynthesis, α: the initial linear slope of photosynthesis *versus* irradiance, E_c_: the light compensation points when net photosynthesis is zero, E_k_: the irradiance for onset of light saturated photosynthesis. From Binzer and Sand-Jensen (2002b)*.

**Table 2:** **Increase (x-fold) of maximum gross production of O_2_ at high (250 μmol L^−^^1^) relative to low (20 μmol L^−^^1^) CO_2_ concentration in alkaline water (5000 μmol L^−^^1^ DIC) of leaves and freshwater plant communities at two densities (LAI; 2 or 10 m^2^ m^−^^2^)**.

	Temperature (°C)	Leaves	Community (LAI 2 m^2^ m^−2^)	Community (LAI 10 m^2^ m^−2^)
**${\text{HOC}}_{\text{3}}^{-}$ USERS**				
*Potamogeton crispus*	15	2.1	1.45	1.16
*Potamogeton pectinatus*	15	2.0	1.33	0.96
**CO_2_ USERS**				
*Callitriche cophocarpa*	15	9.0	3.32	2.46
	25		2.14	1.91
*Sparganium simplex*	15	9.3	4.24	1.91
	25		4.16	1.54

*The ratio GP_max_ (high CO_2_):GP_max_ (low CO_2_) is shown. Leaf values are from Sand-Jensen (1983) and community values from Sand-Jensen et al. (2007)*.

**Figure 4:**
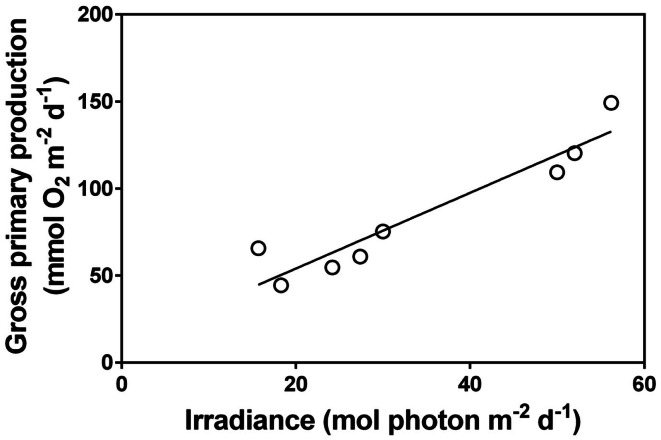
**Relationship between daily surface irradiance and community gross primary production in a shallow pond in Öland, Sweden over a daily time course of 8 days with naturally variable light conditions (each data point represents 1 day)**. The dominant submerged aquatic plant in the pond was, *Chara virgata* (data extracted from Christensen et al., 2013). Pearson correlation = 0.96, *P* < 0.001.

Up scaling of metabolic analyses from communities of submerged aquatic plants to entire ecosystems dominated by rooted plants have only been done in a few instances. Kelly et al. (1983) studied a shallow, densely vegetated stream (Gryde Stream, Denmark) by open water O_2_ measurements and confirmed that incoming irradiance was the main determinant of daily and seasonal variations of underwater photosynthesis which was only light saturated for a few hours at noon on clear summer days. The high CO_2_ concentrations (typically 10-fold air equilibrium) in lowland streams is a prerequisite for the high photosaturated rates and strong light dependency of submerged plants in general and CO_2_ users in particular (Sand-Jensen and Frost-Christensen, 1998). With natural CO_2_ concentrations close to air equilibrium, as observed in most lakes and ponds and in streams in the afternoon after several hours of planktonic photosynthesis (Sand-Jensen and Frost-Christensen, 1998; Christensen et al., 2013), CO_2_ plays a stronger regulatory role for photosynthesis particularly in open plant stands of low self shading (Sand-Jensen and Frost-Christensen, 1998). Moreover, the species rich group of terrestrial plants in lowland streams would be unable to survive if the water had not been greatly supersaturated as their CO_2_ compensation points resemble or exceed the CO_2_ concentrations at air equilibrium (Sand-Jensen and Frost-Christensen, 1999). Recent use of open water measurements of O_2_ and pH in shallow, alkaline ponds dominated entirely by charophytes documents that high biomass densities in late summer are attained by sustained slow growth over the preceding 3 months at very low nutrients concentrations in the water, and that daily photosynthesis is mostly limited by light (Figure 4) and only briefly by DIC at high pH (>9.5), and with virtually no CO_2_ available in the afternoon (Christensen et al., 2013). Only submerged aquatic plants capable of using ${\text{HCO}}_{3}^{-}$ and concentrating CO_2_ internally at the site of Rubisco can thrive in this environment (Sand-Jensen et al., 2010). Plant species forming dense communities in shallow ponds must also be able to tolerate substantial diurnal variations in temperature (e.g., 18–32°C) and O_2_ (hypoxic to twice air equilibrium) (Christensen et al., 2013). Daily photosynthesis and respiration were high in the pond and closely interrelated showing that newly produced organic matter was mostly rapidly respired by plants and bacteria.

Overall, the analyses of individual phytoelements, communities, and ecosystems confirm that the relative roles of light and DIC for determining photosynthesis are closely interrelated and highly dependent on plant density and species affinities for CO_2_ and ${\text{HCO}}_{3}^{-}$. Maximum photosynthetic rates under light and inorganic carbon saturation are quite variable both between and within species depending on selected strategies and the investment in catalytic machinery coupled to supply of resources (e.g., nutrients). While photosaturated photosynthetic rates are strongly dependent on species, acclimation, and temperature, light limited rates are rather temperature independent and relatively similar among species (Frost-Christensen and Sand-Jensen, 1992). As the importance of light limitation for community photosynthesis increases in dense plant stands, the influence of species, temperature, and DIC supply decline and enable us to predict community photosynthesis primarily from the overall distribution and absorptance of light in the canopy (Binzer and Sand-Jensen, 2002a; b; Binzer et al., 2006).

### Recent advances in underwater photosynthesis in terrestrial wetland plants

Terrestrial wetland plants grow in waterlogged soils and/or sediments with shallow standing water, so that a large proportion of the shoot is in contact with air. So, aerial photosynthesis predominates but these plants can experience episodes of complete submergence during floods. Although much more tolerant of submergence than non-wetland terrestrial species, submergence is regarded as a serious abiotic stress for terrestrial wetland plants, but species (and genotypes within a species) differ markedly in submergence tolerance (Bailey-Serres and Voesenek, 2008; Colmer and Voesenek, 2009). The impeded gas exchange under water restricts respiration and photosynthesis (See Challenges Under Water – Reduced Gas Diffusion and Light Penetration); photosynthesis can also be limited by low light when submerged (See Challenges Under Water – Reduced Gas Diffusion and Light Penetration and Underwater Photosynthesis in Submerged Aquatic plants and Recent Advances). Thus, submergence disrupts energy metabolism of terrestrial plant species as a result of a reduced O_2_ supply (at least during the night, in some tissues) and/or diminished carbohydrate status because of the restricted photosynthesis when under water.

Terrestrial wetland species lack most of the adaptive leaf features for inorganic carbon acquisition for photosynthesis as described in Section “Underwater Photosynthesis in Submerged Aquatic Plants and Recent Advances” for aquatic and acclimated amphibious plants. Thus, when compared with leaves of aquatic plants, those of terrestrial plants generally have larger overall apparent resistance to diffusion of CO_2_ from the bulk medium to chloroplasts, so that slow CO_2_ uptake restricts underwater photosynthesis. Underwater photosynthesis by leaves of terrestrial wetland species is lower than that achieved by aquatic species, when compared per unit of leaf dry mass (Sand-Jensen et al., 1992; Colmer et al., 2011).

The few studies available show, however, that the low photosynthesis when under water enhances survival of submerged terrestrial plants (Vervuren et al., 1999; Mommer et al., 2006b; Vashisht et al., 2011). Both the sugars and O_2_ produced would likely contribute to enhanced survival when submerged (Mommer and Visser, 2005), and in the case of sugars especially when submergence lasts more than a few days and internal carbohydrates become depleted. Depletion of carbohydrates during submergence is considered a major factor influencing survival of submerged rice (Setter and Laureles, 1996) and determining recovery following desubmergence and ultimately grain yield in flood-prone areas (Bailey-Serres et al., 2010; Mackill et al., 2012). The O_2_ produced in photosynthesis can travel from leaves to roots via aerenchyma, and so this endogenously produced O_2_ improves the internal aeration of submerged plants (e.g., rice; Pedersen et al., 2009; Winkel et al., 2013).

A recent review (Colmer et al., 2011) highlighted there are few studies of underwater photosynthesis by terrestrial wetland plants, and few of these compared rates underwater with those achieved by leaves in air. Similarly, a quantitative understanding of the potential role of underwater photosynthesis to whole plant carbon budgets during submergence seems to be lacking for terrestrial wetland species, whereas carbon budgets for several aquatic species (e.g., van der Bijl et al., 1989) and systems (e.g., Christensen et al., 2013) have been evaluated. For some terrestrial wetland species, only a few crude leaf level estimates of carbon budgets have been considered (e.g., in Colmer and Pedersen, 2008), but the potential contribution of underwater photosynthesis to carbon gain was demonstrated in growth studies of completely submerged rice, albeit under controlled conditions (e.g., Pedersen et al., 2009). Studies of whole plant carbon budgets in field conditions are generally lacking, even understanding of this aspect for the important wetland crop rice submerged in various field scenarios appears to be incomplete.

Detailed studies of underwater photosynthesis of terrestrial wetland species have focused on production and performance of submergence acclimated leaves. New leaves produced when under water by some terrestrial wetland species are better acclimated for underwater photosynthesis than the aerial leaves (Mommer et al., 2007). The acclimated leaves have a thin cuticle and overall are also thinner and of less breath (Mommer and Visser, 2005). These morphological and anatomical differences as compared with the usual leaves produced in air, reduce the resistance to CO_2_ (and O_2_) diffusion between the bulk medium and chloroplasts in submerged leaves, owing to narrower DBLs (suggested by Colmer et al., 2011), lower cuticle resistance (Mommer et al., 2006b), and shorter internal diffusion path lengths (Mommer et al., 2006a). However, although a reduced cuticle that enhanced underwater gas exchange occurs in several terrestrial wetland species (few species have been evaluated to date), the magnitude of the reduction in apparent resistance to gas exchange with the medium was not correlated with submergence tolerance for the species tested (Mommer et al., 2007), highlighting the need for further experimental investigations.

Some recent work on underwater photosynthesis by submerged terrestrial wetland plants has evaluated the contribution of gas films on superhydrophobic leaf surfaces to gas exchange with floodwaters. Leaf surface hydrophobicity (i.e. water repellence) is a feature that sheds off water in wet aerial environments (Smith and McClean, 1989; Brewer and Smith, 1997) and promotes “self cleansing,” enhancing leaf performance and reputably lowering susceptibility to pathogens (Neinhuis and Barthlott, 1997). Some terrestrial wetland species have super hydrophobic leaves that when submerged retain a gas film, e.g., rice (Raskin and Kende, 1983) and *Phragmites australis* and others (Colmer and Pedersen, 2008). Gas films enhance CO_2_ uptake for underwater photosynthesis (Raskin and Kende, 1983; Colmer and Pedersen, 2008; Pedersen et al., 2009) and O_2_ entry for respiration in darkness (Colmer and Pedersen, 2008; Pedersen and Colmer, 2012). The enhancement by leaf gas films of CO_2_ uptake (in light) and of O_2_ (in darkness) was demonstrated by the marked declines in underwater photosynthesis and respiration when the films were experimentally removed (Colmer and Pedersen, 2008; Pedersen et al., 2009; Pedersen and Colmer, 2012). In addition, leaves produced in air by terrestrial wetland species that did not form gas films when submerged (i.e. leaves of these species were not sufficiently hydrophobic), had lower rates of underwater photosynthesis than those that did form gas films (Colmer and Pedersen, 2008; Colmer et al., 2011). As one example, leaf segments of rice with dissolved CO_2_ set as in a field pond and with underwater photosynthesis measured as described in Section “The Rotating Wheel Incubator,” showed rates four- to fivefold higher for leaf segments with intact gas films compared to those with the films experimentally removed (Winkel et al., 2013). Moreover, a field study using *in situ* monitoring of O_2_ in rhizomes of *Spartina anglica* demonstrated the benefit of having leaf gas films to internal aeration during complete submergence, both during day and night tides (Winkel et al., 2011). Summing up, leaf gas films enhance underwater photosynthesis and internal aeration of some terrestrial wetland plants when submerged, with benefits also demonstrated to growth when submerged in controlled experiments (e.g., rice; Pedersen et al., 2009).

## Underwater Photosynthesis – Approaches and Methods

Conventional infrared gas analyzer (IRGA) systems following CO_2_ exchange in air do not work under water, so dedicated measuring systems are required to quantify underwater net photosynthesis and dark respiration. DIC can be measured by injection of small aliquots of water into concentrated acid in a bubble chamber purged with gaseous N_2_ carrying the released CO_2_ into an IRGA (Vermaat and Sand-Jensen, 1987). However, photosynthesis measurements based on DIC determinations are thus based on discrete measurements at selected times and can be complicated because of the large and variable combined pool of DIC in water (See Underwater Photosynthesis in Submerged Aquatic plants and Recent Advances and The CO_2_ Equilibria in Water). Indirect methods to track DIC changes can be based on continuous measurements of pH in solution (Maberly, 1996). The DIC technique to measure photosynthess has potential errors if: (i) DIC is removed by external carbonate precipitation, (ii) internal DIC accumulates in tissues or colony gels, (iii) DIC dissolution of solid carbonates occurs, or (iv) DIC is released from internal pools (McConnaughey et al., 1994; Sand-Jensen et al., 2009). External measurements of pH to estimate DIC changes have the same potential errors as above and, moreover, also due to direct exchange of protons from tissues not always being closely coupled to DIC exchange. Therefore, most methods for studies of underwater net photosynthesis are based upon O_2_ detection.

In contrast to gas exchange measurements of photosynthesis by leaves in air using open systems and CO_2_ detection, underwater measurements commonly use closed systems and detection of O_2_. In addition to the rationale for O_2_ detection described in the preceding paragraph, O_2_ detection also enables measurements in waters of substantially different DIC concentrations (e.g., softwater lakes up to 100 μmol L^−1^, ocean approximately 2000 μmol L^−1^ and hardwater lakes up to 10000 μmol L^−1^). The drawback of closed systems is that these are non-steady-state (i.e. DIC declining and O_2_ increasing with time). Use of open systems with O_2_ detection is constrained by reliable continuous detection of differences in O_2_ concentrations between incoming and outgoing solutions from an appropriate chamber.

Changes in O_2_ concentration over time are straightforward to measure with Clark type amperometric electrodes or more recently by use of O_2_ sensitive optodes. Oxygen partial pressure (pO_2_) or dissolved O_2_ can be continuously monitored in water with an accuracy of 0.01 kPa or 0.2 μmol L^−1^ (Strickland and Parsons, 1972). Photosynthesis determined from changes in O_2_ and DIC pools dissolved in the surrounding water requires that those are much greater than changes in such pools within the plant tissue (Sand-Jensen and Prahl, 1982). This is best achieved by having large incubation volumes relative to plant volumes. Alternatively, changes in tissue pools can be measured (Sand-Jensen et al., 2005) or be deduced by establishment of true steady state where tissue concentrations remain constant or quasi steady state where tissue concentrations changes proportionally to external concentrations (Sand-Jensen and Prahl, 1982). Measurements of underwater photosynthesis based upon O_2_ evolution can include great error when plants with highly porous tissues (perhaps variable in volume and having much higher “solubility” of O_2_ than water; See Medium and Tissue) are incubated in small chambers (Hartman and Brown, 1967; Richardson et al., 1984). On the other hand, measurements of underwater photosynthesis based upon changes in DIC can include extreme error when plant tissues (or colony matrices in the case of algae and cyanobacteria) hold very large pools of DIC that do not change in concert with those in the surrounding water. For example, DIC in the colony gel of *Nostoc zetterstedtii* continues to support photosynthesis after water pools have been exhausted, and in darkness respiratory CO_2_ replenishes this internal pool before being released to the water (Sand-Jensen et al., 2009).

Measurements of radioactive labeling of the DIC pool with ^14^C and the use of pulse amplitude modulated (PAM) fluorometry are also methods to measure photosynthetic performance under water; these technique are beyond the focus of the present paper so readers are referred to e.g., Adams et al. (1978) or Kemp et al. (1986) for methods on ^14^C and to Silva et al. (2009) or Suggett et al. (2011) and chapters therein for PAM approaches.

### The CO_2_ equilibria in water

Understanding the chemistry of dissolved DIC and the proportional changes in its three constituents (CO_2_, ${\text{HCO}}_{3}^{-}$ and ${\text{CO}}_{3}^{2-}$) depending on ionic strength, temperature, and primarily pH (Mackereth et al., 1978) is essential because it determines the availability of the preferred CO_2_ source and the supplementary ${\text{HCO}}_{3}^{-}$ source for underwater net photosynthesis. When CO_2_ dissolves in water, the following equilibrium is established:

(4) $$\text{C}{\text{O}}_{2\left(\text{aq}\right)}+{\text{H}}_{2}\text{O}↔\left({\text{H}}_{2}\text{C}{\text{O}}_{3}\right)↔{\text{HCO}}_{3}^{-}+{\text{H}}^{+}↔{\text{CO}}_{3}^{2-}+2{\text{H}}^{+}$$

**Figure 5:**
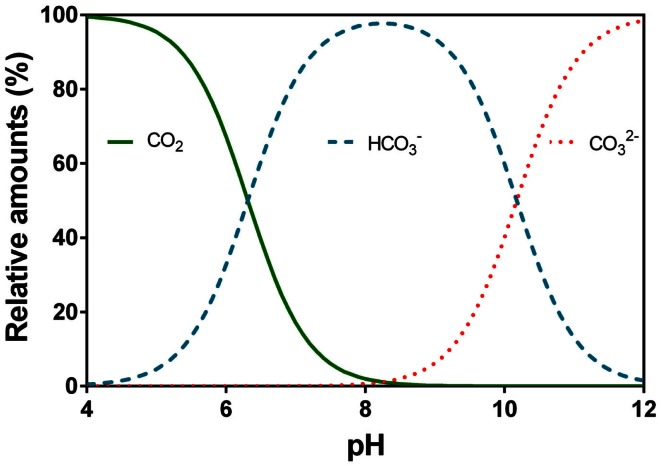
**Relative speciation (%) of carbon dioxide (CO_2_), bicarbonate (${\text{HCO}}_{3}^{-}$), and carbonate (${\text{CO}}_{3}^{2-}$) in water as a function of pH**. The example given is at 20°C and electrical conductivity of 250 μS cm^−1^. Data were calculated using Gutz (2012) with the apparent *p*K_1_ = 6.532 and *p*K_2_ = 10.329 (Schwarzenbach and Meier, 1958).

CO_2_’s reaction with water (H_2_O) forming carbonic acid (H_2_CO_3_) is a time dependent process which in some organisms is catalyzed by the enzyme carbonic anhydrase. H_2_CO_3_ can dissociate immediately into a proton (H^+^) and bicarbonate (${\text{HCO}}_{3}^{-}$) so the dissolution of CO_2_ into water causes pH to drop. At high pH, ${\text{HCO}}_{3}^{-}$ can further dissociate into a second H^+^ and carbonate (${\text{CO}}_{3}^{2-}$). The relative distribution of the three main inorganic carbon species with pH is shown (Figure 5). The pKa_1_ is 6.532 and is referred to as the apparent pKa_1_ as only little CO_2_ is converted into carbonic acid (hence the brackets in Eq. 4) while the majority remains in solution as CO_2_(aq) also referred to as free CO_2_; pKa_2_ is 10.329 (Schwarzenbach and Meier, 1958; Stumm and Morgan, 1996; Gutz, 2012). Below pH 6, most of the DIC is present as CO_2_, which is usually more readily used for underwater photosynthesis than ${\text{HCO}}_{3}^{-}$. Between pH 7 and 10, ${\text{HCO}}_{3}^{-}$ dominates, a carbon species that can be used as an additional carbon source among species in most taxonomic groups of aquatic plants except for pteridopytes and mosses (Raven and Hurd, 2012). Only at pH higher than 10, a significant proportion of the DIC is in the form of ${\text{CO}}_{3}^{2-}$ which apparently is not taken up by any phototrophs in ionic form but can perhaps be made available in acid zones on plant surfaces by back titration with released protons (conversion toward the left in Eq. 4).

In freshwaters and seawater, the alkalinity (sum of alkaline ions buffering added H^+^; units in mequiv. L^−1^ or mmol L^−1^ for monovalent ${\text{HCO}}_{3}^{-}$ in water which is in air equilibrium of negligible OH^−^ and ${\text{CO}}_{3}^{2-}$) is almost entirely controlled by the carbonate systems with insignificant contribution from silicate and phosphate, and with some contribution by borate in seawater. At pH above 9, OH^−^ has a significant contribution to alkalinity being 0.074 mmol L^−1^ at pH 10 and 0.74 mmol L^−1^ at pH 11 at an alkalinity of 2 mmol equivalents L^−1^ (Table 3). It is thus convenient to distinguish between the total alkalinity (TA) and the CA (Dickson, 1981; Stumm and Morgan, 1996). The chemical species contributions to the two alkalinities are:

(5) $$\begin{array}{lll} \text{CA} & ={\left(\text{HC}{\text{O}}_{\text{3}}\right)}^{-}\text{ + 2}{\left(\text{C}{\text{O}}_{\text{3}}\right)}^{2-} & \end{array}$$

(6) $$\begin{array}{lll} \text{TA} & =\text{CA}+{\left({\text{H}}_{2}{\text{BO}}_{3}\right)}^{-}\text{ + 2}{\left(\text{HB}{\text{O}}_{\text{3}}\right)}^{\text{2}-}\text{ + }{\left(\text{OH}\right)}^{-}-{\left(\text{H}\right)}^{\text{ + }} & \end{array}$$

**Table 3:** **Distribution of DIC, CO_2_, ${\text{HCO}}_{3}^{-}$, ${\text{CO}}_{3}^{2-}$, and OH^−^ as a function of pH at constant total alkalinity of 2 mmol H^+^ equivalents L^−^^1^ at 20°C**.

pH	DIC (mmol L^−1^)	CO_2_ (mmol L^−1^)	${\text{HCO}}_{3}^{-}$ (mmol L^−1^)	${\text{CO}}_{3}^{2-}$ (mmol L^−1^)	OH^−^ (mmol L^−1^)
6.00	6.5073	4.5074	1.9998	0.0001	0.0000
6.05	6.0170	4.0172	1.9997	0.0001	0.0000
6.10	5.5801	3.5803	1.9997	0.0001	0.0000
6.15	5.1907	3.1909	1.9997	0.0002	0.0000
6.20	4.8436	2.8438	1.9996	0.0002	0.0000
6.25	4.5343	2.5345	1.9996	0.0002	0.0000
6.30	4.2586	2.2588	1.9995	0.0002	0.0000
6.35	4.0128	2.0131	1.9995	0.0002	0.0000
6.40	3.7938	1.7941	1.9994	0.0003	0.0000
6.45	3.5986	1.5990	1.9994	0.0003	0.0000
6.50	3.4246	1.4250	1.9993	0.0004	0.0000
6.55	3.2696	1.2700	1.9992	0.0004	0.0000
6.60	3.1313	1.1318	1.9991	0.0004	0.0000
6.65	3.0082	1.0087	1.9990	0.0005	0.0000
6.70	2.8983	0.8989	1.9988	0.0006	0.0000
6.75	2.8004	0.8011	1.9987	0.0006	0.0000
6.80	2.7132	0.7139	1.9985	0.0007	0.0000
6.85	2.6354	0.6362	1.9984	0.0008	0.0001
6.90	2.5661	0.5670	1.9982	0.0009	0.0001
6.95	2.5042	0.5053	1.9979	0.0010	0.0001
7.00	2.4491	0.4503	1.9977	0.0011	0.0001
7.05	2.3999	0.4013	1.9974	0.0012	0.0001
7.10	2.3561	0.3576	1.9971	0.0014	0.0001
7.15	2.3169	0.3186	1.9968	0.0016	0.0001
7.20	2.2820	0.2839	1.9964	0.0018	0.0001
7.25	2.2509	0.2530	1.9959	0.0020	0.0001
7.30	2.2230	0.2254	1.9954	0.0022	0.0001
7.35	2.1982	0.2008	1.9949	0.0025	0.0002
7.40	2.1760	0.1789	1.9942	0.0028	0.0002
7.45	2.1561	0.1594	1.9935	0.0031	0.0002
7.50	2.1383	0.1420	1.9927	0.0035	0.0002
7.55	2.1223	0.1265	1.9919	0.0039	0.0003
7.60	2.1080	0.1127	1.9909	0.0044	0.0003
7.65	2.0951	0.1004	1.9898	0.0050	0.0003
7.70	2.0835	0.0894	1.9885	0.0056	0.0004
7.75	2.0730	0.0796	1.9871	0.0062	0.0004
7.80	2.0635	0.0709	1.9856	0.0070	0.0005
7.85	2.0548	0.0632	1.9838	0.0078	0.0005
7.90	2.0469	0.0562	1.9819	0.0088	0.0006
7.95	2.0396	0.0501	1.9797	0.0098	0.0007
8.00	2.0328	0.0446	1.9772	0.0110	0.0007
8.05	2.0265	0.0397	1.9745	0.0123	0.0008
8.10	2.0205	0.0353	1.9714	0.0138	0.0009
8.15	2.0149	0.0314	1.9680	0.0155	0.0010
8.20	2.0094	0.0279	1.9641	0.0173	0.0012
8.25	2.0041	0.0248	1.9598	0.0194	0.0013
8.30	1.9989	0.0221	1.9550	0.0217	0.0015
8.35	1.9937	0.0196	1.9497	0.0243	0.0017
8.40	1.9884	0.0174	1.9437	0.0272	0.0019
8.45	1.9830	0.0155	1.9371	0.0304	0.0021
8.50	1.9774	0.0138	1.9297	0.0340	0.0023
8.55	1.9716	0.0122	1.9214	0.0380	0.0026
8.60	1.9655	0.0108	1.9122	0.0424	0.0029
8.65	1.9590	0.0096	1.9020	0.0474	0.0033
8.70	1.9520	0.0085	1.8907	0.0528	0.0037
8.75	1.9445	0.0075	1.8781	0.0589	0.0041
8.80	1.9364	0.0067	1.8642	0.0656	0.0047
8.85	1.9277	0.0059	1.8489	0.0729	0.0052
8.90	1.9182	0.0052	1.8319	0.0811	0.0059
8.95	1.9079	0.0046	1.8133	0.0901	0.0066
9.00	1.8967	0.0040	1.7928	0.0999	0.0074
9.05	1.8846	0.0036	1.7703	0.1107	0.0083
9.10	1.8714	0.0031	1.7457	0.1225	0.0093
9.15	1.8570	0.0027	1.7189	0.1353	0.0104
9.20	1.8415	0.0024	1.6898	0.1493	0.0117
9.25	1.8246	0.0021	1.6582	0.1643	0.0131
9.30	1.8065	0.0018	1.6241	0.1806	0.0147
9.35	1.7870	0.0016	1.5874	0.1981	0.0165
9.40	1.7661	0.0014	1.5480	0.2167	0.0185
9.45	1.7438	0.0012	1.5061	0.2366	0.0208
9.50	1.7201	0.0010	1.4615	0.2576	0.0233
9.55	1.6950	0.0009	1.4144	0.2797	0.0262
9.60	1.6686	0.0008	1.3649	0.3028	0.0294
9.65	1.6408	0.0007	1.3132	0.3269	0.0329
9.70	1.6118	0.0006	1.2594	0.3518	0.0370
9.75	1.5817	0.0005	1.2039	0.3773	0.0415
9.80	1.5506	0.0004	1.1469	0.4033	0.0465
9.85	1.5186	0.0003	1.0887	0.4295	0.0522
9.90	1.4858	0.0003	1.0297	0.4559	0.0586
9.95	1.4525	0.0002	0.9703	0.4820	0.0657
10.00	1.4188	0.0002	0.9109	0.5077	0.0738
10.05	1.3847	0.0002	0.8519	0.5327	0.0828
10.10	1.3505	0.0001	0.7936	0.5568	0.0929
10.15	1.3162	0.0001	0.7364	0.5797	0.1042
10.20	1.2820	0.0001	0.6807	0.6012	0.1169
10.25	1.2478	0.0001	0.6267	0.6211	0.1312
10.30	1.2138	0.0001	0.5747	0.6391	0.1472
10.35	1.1800	0.0001	0.5249	0.6550	0.1651
10.40	1.1462	0.0000	0.4776	0.6686	0.1853
10.45	1.1124	0.0000	0.4327	0.6797	0.2079
10.50	1.0786	0.0000	0.3905	0.6881	0.2333
10.55	1.0446	0.0000	0.3508	0.6937	0.2617
10.60	1.0101	0.0000	0.3138	0.6963	0.2937
10.65	0.9750	0.0000	0.2794	0.6956	0.3295
10.70	0.9389	0.0000	0.2475	0.6914	0.3697
10.75	0.9017	0.0000	0.2181	0.6835	0.4148
10.80	0.8628	0.0000	0.1910	0.6718	0.4654
10.85	0.8220	0.0000	0.1662	0.6558	0.5222
10.90	0.7788	0.0000	0.1435	0.6353	0.5859
10.95	0.7327	0.0000	0.1228	0.6099	0.6574
11.00	0.6831	0.0000	0.1039	0.5792	0.7377

Calculated from Mackereth et al. (1978) and Gutz (2012). See Figure 5 for the relative distribution between CO_2_, ${\text{HCO}}_{3}^{-}$ and ${\text{CO}}_{3}^{2-}$*versus* pH. The yellow highlight refers to Example 1, Section “Medium and Tissue.”

Purging an aqueous solution with pure CO_2_ alters the CA through the addition of ionic carbon species and also through pH related shifts in the partitioning of carbon species already present in the solution (Eqs 4 and 5). However, the TA is not affected by bubbling with CO_2_ as every negatively charged ion is balanced by a proton (Eq. 6). For example, water fresh from the tap often contains CO_2_ above air equilibrium and so bringing it to equilibrium by purging with atmospheric air would thus lower pCO_2_ until a new equilibrium has been reached. According to Eq. 5, CA would decrease slightly as both ${\text{CO}}_{3}^{2-}$ and ${\text{HCO}}_{3}^{-}$ decrease equivalent to the rise of OH^−^ and pH.

For experimental purposes, an aqueous photosynthesis solution is usually prepared with a certain amount of DIC and then pH is adjusted with acid or base to that required to achieve the desired concentration of “free” (i.e. dissolved) CO_2_ and ${\text{HCO}}_{3}^{-}$. Table 3 lists the relationship between pH and amounts of CO_2_, ${\text{HCO}}_{3}^{-}$, ${\text{CO}}_{3}^{2-}$, and OH^−^ at 20°C and a fixed TA, calculated from Gutz (2012). The examples provided in the sections below demonstrate how to apply all the above information in practice.

In the next sections (See “The Rotating Wheel Incubator” to “The Open Natural System”) we describe methods in use for measurements of underwater photosynthesis. The methods scale from phytoelements to communities. The approaches involve laboratory and field techniques and so have different levels of control of key environmental variables influencing photosynthesis.

### The rotating wheel incubator

Principle: Leaf samples or algal thalli are incubated in glass vials of a known concentration of CO_2_ in an aqueous medium, and the sealed vials of known volume are rotated on an incubator under well defined light and temperature conditions. O_2_ produced during incubation is measured by an electrode/optode and underwater net photosynthesis can be calculated based on e.g., leaf area, fresh mass, dry mass, and/or chlorophyll. Alternatively, consumption of DIC can be used as a photosynthetic measure. Incubation in darkness provides data on dark respiration.

#### Medium and tissue

The choice of medium is basically between an artificial medium with a well defined ion and gas composition or ambient water with the ion and gas composition of natural habitats (essential chemical parameters such as pH, DIC, and alkalinity should be characterized). An example of an artificial medium is the Smart and Barko (1985) general purpose culture medium. This medium contains (mmol L^−1^) 0.62 Ca^2+^, 0.28 Mg^2+^, 0.28 SO_4_^2−,^ and 1.24 Cl^−^ and KHCO_3_ (sometimes mixed with NaHCO_3_) is used to generate the required DIC. HCl, NaOH (or KOH), atmospheric air or gas mixtures of known pCO_2_ can be used to adjust pH to a required value based on the desired amount of free CO_2_. Since all incubations are short term, there are no micro nutrients or vitamins in this medium. Some studies have also used submergence solutions or “ambient” water from streams or lakes in order to establish a rate of photosynthesis under specific conditions (Sand-Jensen et al., 1992; Nielsen, 1993; Sand-Jensen and Frost-Christensen, 1998) and these can also be adjusted to predefined pH, CO_2_ and/or O_2_ levels. Any production of O_2_ by microalgae or consumption by microbial organisms in ambient water is accounted for in the blanks; micro-filtration of water is commonly used to remove background microflora.

Photorespiration, as previously demonstrated for rice (Setter et al., 1989) and the aquatic pteridophyte, *Isoetes australis*, (Pedersen et al., 2011), during incubation is a potential issue as the evolved O_2_ is trapped in solution of the closed glass vial. The risk of photorespiration is increased during experiments at high temperature (Long, 1991) and with very low DIC and CO_2_ concentrations leading to low ratios of CO_2_ to O_2_ at the site of Rubisco (Maberly and Spence, 1989; Sand-Jensen and Frost-Christensen, 1999). Therefore, the starting partial pressure of O_2_ (pO_2_) should be brought down to approximately 50% of air equilibrium, i.e., 10 kPa. This is sufficient to address the issue of photorespiration (provided that incubation do not last long periods so that O_2_ produced increases above air equilibrium) and at the same time there is still enough O_2_ in the medium to prevent tissue anoxia before photosynthesis starts producing O_2_ (Colmer and Pedersen, 2008). In practice, equal volumes of medium (including all ions) are bubbled with either air or N_2_. After mixing the two solutions, the pO_2_ will be approximately 10 kPa and ${\text{HCO}}_{3}^{-}$ can be added to the medium and pH adjusted accordingly to achieve the desired amount of free CO_2_ (see example below).

In some situations, an organic buffer may be used to maintain a constant pH in the medium during incubation. In practice, however, ${\text{HCO}}_{3}^{-}$ is a natural and often sufficient buffer in itself and we do not recommend using buffers if the CA is above 1 mmol L^−1^ as ${\text{HCO}}_{3}^{-}$ would be sufficient to buffer against large pH fluctuations during incubation (Sand-Jensen et al., 1992; Colmer and Pedersen, 2008). Moreover, organic buffers can also modify membrane porters and pH at plant surfaces modifying ${\text{HCO}}_{3}^{-}$ use and influx/efflux of CO_2_ (Price and Badger, 1985; Larsson and Axelsson, 1999; Moulin et al., 2011). pH of the medium should be measured in a sample taken of the initial solution and then also in vials after incubations. With the ongoing advancement of optodes, pH may even be measured without opening the vials if applying pH sensitive microdots (See “O_2_ Measurements” for description of O_2_ sensitive microdots). If additional buffering is required, i.e. pH measurements after incubation show unacceptable drift in pH, then MES or TES buffers may be used, e.g., at a concentration of 5 mmol L^−1^ (Pedersen et al., 2009, 2010), though the possible influence of these buffers on ${\text{HCO}}_{3}^{-}$ use must be kept in mind.

The vials (10–100 mL glass vials with ground glass stoppers) are filled with medium using a siphon. By siphoning the medium into the bottom of each vial, exchange of O_2_ and particularly CO_2_ with the atmosphere is minimized; prepare sufficient medium to flush the vials at least twice the volume, and fill the vials completely. An air bubble can hold 36-fold more O_2_ as the same volume of deionized (DI) water at 25°C, so bubbles in the vials introduce significant error to the net photosynthesis measurements. A set of vials without tissue serves as blanks and is incubated along with the vials containing tissue samples in the rotating incubator. The blanks serve to provide the starting pO_2_ in the vials and also to correct for any O_2_ production or consumption (e.g., by algae, bacteria, or chemical processes) if ambient water is used as medium. Glass beads (Ø = 3–5 mm; two in each 25 mL vial) are added to each vial to provide mixing as the vials are rotating in the incubator.

The amount of tissue added to each vial depends on the activity of the tissue, the amount of DIC and free CO_2_, the light level (PAR), and the temperature. At saturating light and CO_2_ levels and at 25°C, 0.5 mg fresh mass mL^−1^ medium is often a good choice as this will result in a rise of pO_2_ by approximately 2–5 kPa within an hour of incubation providing reproducible and accurate determination of O_2_ regardless of the technique employed (see below). However, both microelectrodes and optodes have a resolution of approximately 0.01 kPa so a change in 1 kPa could also be sufficient. At lower CO_2_ and/or light levels, more tissue may be required or alternatively, longer incubation times are needed. However, small tissue samples are preferred to prevent self shading and to promote good mixing in the vials so that tissues are well exposed to light and chemicals during incubation.

Example 1: Preparation of artificial floodwater with CA of 2.0 mmol L^−1^ and 200 μmol free CO_2_ L^−1^. Prepare a solution of DI water containing Ca^2+^, Mg^2+^, SO_4_^2−^, and Cl^−^ at the concentrations described above. Divide the solution into two containers and bubble one half of the solution with air and the other half with N_2_ for 20 min and then mix the two solutions. Add the required amount of DIC (Table 3, highlighted in yellow for this example) which is 2.2 mmol L^−1^. Add the DIC in the form of KHCO_3_, NaHCO_3_ or a mixture, and acidify the solution to pH 7.35 using HCl. This results in a solution with a CA of 2 mmol L^−1^ (in mmol L^−1^: 1.995 ${\text{HCO}}_{3}^{-}$ + 0.002 ${\text{CO}}_{3}^{2-}$) and 200 μmol L^−1^ CO_2_ (Table 3).

#### Incubator with light and temperature control

The incubator provides constant temperature and mixing throughout the incubation. It consists of a vertically rotating wheel where glass bottles or vials can be clipped on facing the light source. The wheel rotates at about 10 rpm in a tank with temperature controlled water and a transparent glass or Perspex wall for illumination at various irradiances (Figure 1C).

The rotating wheel incubator was originally invented for photosynthesis measurements in phytoplankton (Steemann Nielsen, 1952) and the typical light source in commercially available models consists of a rack of fluorescent tubes. However, it is hard to achieve PAR levels much above 500 μmol photons m^−2^ s^−1^ with fluorescent light so high pressure metal halide lamps (mercury or sodium) or light emitting plasma lamps are required to provide the levels of light needed to light saturate net photosynthesis by leaves of many terrestrial species and some macroalgae with thick thalli.

Photosynthesis *versus* light curves (i.e. light response curves) are obtained by: (i) regulating light intensities by varying the distance of the light source to the incubator, (ii) placing neutral shading filters in front of the light source, (iii) placing a box with neutral shading filters of variable transmission in front of individual vials, or (iv) by wrapping the vials in layers of neutral shading mesh, or by a combination of these various approaches.

#### O_2_ measurements

The O_2_ produced or consumed during incubation can be measured directly in the glass vials using O_2_ electrodes or optodes. In the absence of good electrodes or optodes, the Winkler titration can also be applied; see Strickland and Parsons (1972) for details.

Contemporary methods for O_2_ measurements in water involve either Clark type electrodes or optodes. A Clark type O_2_ electrode measures pO_2_ as molecular O_2_ transverses a membrane before the electrochemical reaction on the cathode results in a current which is linearly proportional to the pO_2_ of the medium. Since the electrode consumes O_2_, a conventional large O_2_ electrode is quite stirring sensitive and it is thus much more convenient to use an O_2_ microelectrode which consumes little O_2_ to address the stirring issue during measurements; O_2_ microelectrodes can have a stirring sensitivity of less than 1% (Revsbech and Jørgensen, 1986; Revsbech, 1987). Oxygen microelectrodes typically have a temperature coefficient of approximately 1–3%°C^−1^ (Revsbech, 1987; Gundersen et al., 1998) so temperature control during measurements is essential. The temperature effect on electrodes (and optodes, see below) is primarily caused by changes in diffusion and electrochemistry. In addition, temperature also influences solubility of gases, and metabolic rate of the tissues.

The measuring principle of optodes is quite different from that of a Clark type electrode. In the optode, light excites a fluorophore coated onto the tip of fiber optics and the excited light is subsequently transmitted back and measured by a spectroradiometer (Klimant et al., 1997). Alternatively, the fluorophore can be coated onto tiny plastic patches which (microdots) can be mounted directly in the medium where O_2_ is to be measured; the microdot with the fluorophore is then illuminated from the outside through the transparent wall of the container. Molecular O_2_ quenches the florescence so that the transmitted signal can be calibrated toward O_2_ in the medium; the relationships between quenching and pO_2_ is non-linear. Optodes do not consume O_2_ and are thus completely insensitive to stirring. However, O_2_ optodes can have higher temperature coefficients than Clark type microelectrodes and require even better temperature control during measurements (Kragh et al., 2008). On the other hand, optodes can be built into the individual glass vials (microdots glued onto the glass wall inside the vial) and the O_2_ concentration can be measured in a non-destructive manner (Kragh et al., 2008). The great advance of this approach is that vials can remain sealed and be returned to the rotating wheel if a preliminary reading shows that longer incubation is required in order to obtain the necessary accuracy, or O_2_ evolution can be followed over time to ensure *quasi* steady state measurements or to elucidate possible temporal patterns.

#### Supporting measurements and calculations

**Figure 6:**
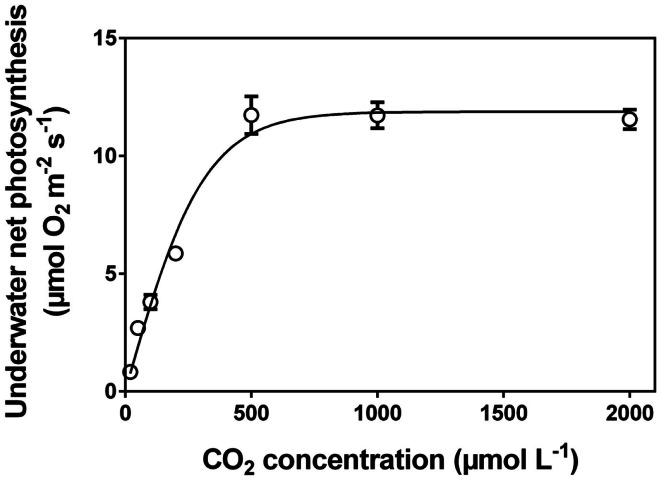
**Underwater net photosynthesis *versus* CO_2_ concentration in the medium for excised leaf segments of *Hordeum marinum***. Leaf segments (30 mm) were incubated in 35 mL glass vials with various well defined CO_2_ concentrations on a rotating wheel with PAR of 350 μmol photons m^−2^ s^−1^ at 20°C (see Figure 1C). O_2_ evolution was measured with a Clark type O_2_ microelectrode and underwater net photosynthesis was calculated as O_2_ evolution per projected area per unit time (See “The Rotating Wheel Incubator”). Data (mean ± SE, *n*=5) from (Pedersen et al., 2010). Note: leaves of *H. marinum* are superhydrophobic and so possess a gas film when underwater.

After measuring O_2_ of each vial, the tissue must be processed according to standard procedures to establish the area, the fresh mass or dry mass, the chlorophyll concentration, or all of the above. The underwater net photosynthesis is calculated as the net O_2_ evolution rate per unit tissue per unit time. In practice, the change in O_2_ content in each vial (change in O_2_ concentration multiplied by the volume of the vial; individual volumes of vials (i.e. minus the volume of the glass beads, etc.) must be established) divided by the incubation time and divided by the amount of tissue (i.e., mass, area or any other of the above mentioned parameters used to scale photosynthesis per sample unit). An example of a CO_2_ response curve established with the technique described here in Section “The Rotating Wheel Incubator” is shown in Figure 6.

### The closed chamber with injection ports

Principle: a leaf or algal thalli sample is incubated in a closed chamber with internal mixing and possessing injection ports and fitted with an electrode/optode that follows O_2_ concentration. The amount of free CO_2_ can be manipulated by injection of acid or base while a fitted pH electrode allows calculation of the exact CO_2_ level. The approach enables production of a complete light or CO_2_ response curve based on the same sample, and underwater net photosynthesis can be calculated based on e.g., leaf area, fresh mass, dry mass, and/or chlorophyll concentration. Incubation in darkness can provide data on dark respiration.

#### Chamber with light and temperature control

The chamber for measurements of underwater net photosynthesis enables measurements with light, temperature, and CO_2_ manipulations in water, with monitoring of O_2_ with time. Chambers are commercially available for underwater photosynthesis measurements on macro algae, phytoplankton, or isolated chloroplasts and these are made from glass, acrylic glass, or acetal. These chambers can also be custom built to match specific electrodes, light sources, and fitted with extra ports for temperature and PAR sensors and injection of acid/bases or inhibitors. The chamber must be made from a material the can be sterilized and also have a least one transparent side to enable illumination of the sample. The light source can be diode based (650 nm red diode) or “full spectrum” halogen light to simulate white sunlight. Pay attention to the fact that some lighting devices are unable to produce sufficient light to saturate the photosynthesis of some terrestrial leaves or thick macroalgae thalli. Illumination (even by means of fiber optics) produces heat, so cooling of the chamber by a water jacket is crucial.

A light sensor small enough to measure inside the chamber is also essential. The spherical PAR sensor US-SQS/L (Walz, Effeltrich, Germany) is of a size (Ø = 3.7 mm) that enables permanent installation in most chambers.

Finally, the issue of mixing must be addressed. The simplest solution is to use a glass coated stir bar (avoid Teflon coated stir bars as these can hold O_2_) which is isolated from the sample with a coarse mesh to prevent contact with the tissue. It may be necessary to fix the tissue in the swirling current; if the tissue rotates with the water current in the chamber, the DBL will be larger than if the tissue is fixed. The thicker DBL increases the apparent resistance to CO_2_ uptake or O_2_ escape.

#### O_2_ and pH measurements

O_2_ measurements in the closed chamber are similar to O_2_ measurements in the vials described in Section “O_2_ Measurements.” An O_2_ sensor (Clark type electrode or optode) is fixed in the chamber in one of the ports, or if an optode is used, a patch with fluorophore can be glued onto the interior wall. A pH electrode is fitted in a second port and the signals from both sensors are logged onto a computer with data acquisition software. Calibration of both O_2_ and pH sensors should be performed in the chamber to avoid stirring related artifacts to the calibrations. Remember to pay extra attention to temperature if using O_2_ optodes. It may take a while for the temperature of the solution inside the chamber to equilibrate with that of the cooling jacket, and working in a constant temperature room or keeping solutions in a thermostated water bath will significantly reduce the time it takes before a temperature steady state is obtained; always measure temperature directly in the chambers. Temperature influences electrode or optode performance, solubility of gases, and metabolic rate of the tissues (see Section “O_2_ Measurements”). After insertion of tissue and filling of the chamber with medium (see below), pH can be manipulated by injection of small amounts of acid or base through one of the injection ports. Fit a 27G needle in one of the injection ports and let it function as “over pressure valve” to prevent pressurization during injection of acid or base (or inhibitors); the needle may be left in the stopper during the experiment as diffusion of gases in water is too slow to result in experimental artifacts.

As described in Section “The Rotating Wheel Incubator” for incubations of tissues in closed vials on the wheel, substantial photorespiration can occur if O_2_ is allowed to build up in the medium. Therefore, the susceptibility to photorespiration should initially be established for each tissue type. The linearity of O_2_ production with increasing external pO_2_ is easily tested the following way: a medium with total DIC of 5.0 mmol L^−1^ is prepared from KHCO_3_ in a 5.0 mmol L^−1^ TES buffer adjusted to pH 8.00 and with a pO_2_ of 10 kPa. The tissue is then allowed to photosynthesize up to a pO_2_ of 30 kPa. Here, approximately 500 μmol O_2_ has been produced from 500 μmol CO_2_ and because of the TES buffer the pH has remained at 8.0. Although the DIC pool has declined to 4.5 mmol L^−1^, free CO_2_ has changed by only 10% from 110 to 100 μmol L^−1^. If the O_2_ evolution occurs linearly in this range, it means that the approximately threefold lower CO_2_:O_2_ in the medium, with likely even greater changes in internal CO_2_:O_2_, has not increased photorespiration. If the curve exhibits a saturation tendency (i.e. declining rate of net O_2_ production with increasing pO_2_), photorespiration has probably increased with increasing pO_2_ in the chamber.

Medium and tissue may be prepared as described in Section “Medium and Tissue.” However, as a CO_2_ response curve in the closed photosynthesis chamber often involves conversion of ${\text{HCO}}_{3}^{-}$ into free CO_2_ (dissolved), e.g., by manipulation of pH, enough ${\text{HCO}}_{3}^{-}$ must initially be present in the medium to produce the required levels of free CO_2_. Following injection of small amounts of acid or base to manipulate free CO_2_, the rate of net photosynthesis changes accordingly so that a new rate of net O_2_ production (slope of dissolved O_2_ with time) is established at each dissolved CO_2_. However, pH may also change slightly in the time interval because CO_2_ is extracted from the system as it is fixed via photosynthesis (Eqs 1 and 4). Hence, for every rate of underwater net photosynthesis determined in a time interval, the mean CO_2_ concentration must be calculated in order to present the CO_2_ response curve of the tissue.

Example 2: average free CO_2_ concentration in the pH range from 7.25 to 7.30 in a medium with total DIC of 2.0 mmol L^−1^. According to Gutz (2012), CA of such a solution at pH 7.25 would be 1.77 mmol L^−1^ having 223 μmol CO_2_ L^−1^; at pH 7.30 CA would be 1.80 mmol L^−1^ and have 203 μmol CO_2_ L^−1^. Consequently, the average free CO_2_ concentration in the pH range was 213 μmol CO_2_ L^−1^.

After each experiment, the incubated tissue must be characterized to enable calculation of underwater net photosynthesis rates; the supporting measurements are as those described in section “Supporting Measurements and Calculations.”

### pH drift approach to establish CO_2_ compensation points

Principle: Leaf or algal thalli samples are incubated in glass vials for 16–18 h where after pH and CA or DIC are measured. CO_2_ compensation points and carbon extraction capacity of tissues can be calculated. The method is also used as a diagnostic test for bicarbonate (${\text{HCO}}_{3}^{-}$) use in underwater photosynthesis.

These long term incubations are used to test how far net photosynthesis of a given plant sample at saturating light can extract DIC, i.e. to deplete CO_2_ and ${\text{HCO}}_{3}^{-}$ and drive up pH. Because the objective is to determine the ultimate DIC extraction capacity and maximum upper pH in a standardized way, all incubation vials are prepared to have an equal standard DIC concentration (usually 1–2 mmol L^−1^ for alkaline waters and 0.1–0.3 mmol L^−1^ for softwaters) and a pH, CO_2_, and O_2_ concentration corresponding to air equilibrium (Sand-Jensen et al., 1992, 2009). Artificial media and natural waters can be applied. However, to minimize O_2_ build up and the risk of photorespiration during extended incubation the initial O_2_ can be reduced to 20–50% of air equilibrium. To ensure the maximum possible DIC depletion, the amount of plant material is typically three times larger than in the incubations described in Sections “The rotating Wheel Incubator” and “The Closed Chamber with Injection Ports” though it must still be able to move freely in the vials to ensure adequate mixing.

The initial and final DIC and pH must be determined in order to calculate the DIC extraction capacity during incubation and the CO_2_ compensation point after incubation. Provided no internal pools of DIC and protons interfere with conditions in the water/medium and no precipitation or dissolution of carbonates takes place, DIC can be determined in the medium from CA, pH, temperature, and ionic strength; CA in turn can be determined by acidimetric titration (Stumm and Morgan, 1996). The risk of carbonate precipitation is small in artificial media of KHCO_3_ and much larger in natural waters and artificial media where Ca(HCO_3_)_2_ dominates, the reason being that K_2_CO_3_ is highly soluble and CaCO_3_ is poorly soluble. Calcium carbonate precipitation is likely to take place in pH drift experiments where final pH exceeds 10. Therefore, it is always recommended to directly measure DIC. This can be done by injecting of small water samples into concentrated acid in a bubble chamber purged with N_2_ gas carrying the released CO_2_ into an IRGA (Vermaat and Sand-Jensen, 1987). Water samples may need to be filtered (with no atmospheric contact) if minute CaCO_3_ crystals have been formed in the external water of high pH. It is generally recommendable to determine (or check) CO_2_ compensation points by depletion experiments in media of low initial DIC (<50 μmol L^−1^) and low pH (<6.5) where the interference by ${\text{HCO}}_{3}^{-}$ is low and CaCO_3_ is not formed.

The pH drift technique has also been used to determine DIC consumption at intervals during the ongoing drift of pH upwards (Maberly and Spence, 1983; Spence and Maberly, 1985). DIC, pH, the proportion of carbon species and O_2_ change during the time of incubation. Because all parameters may influence photosynthesis, and exchange with internal DIC and proton pools in the incubated tissue may interfere with calculations, we cannot recommend the procedure for determining rates of net photosynthesis considering the much more accurate and straightforward methods being available today (as described in this review).

### Community photosynthesis in large chambers

Principle: Community photosynthesis is measured in large closed chambers with linear dimensions of 0.5–0.6 m, or larger, to minimize edge effects and make certain that natural changes of plant density, tissue capacity and irradiance through the canopy are maintained. Photosynthetic rates are measured by O_2_ and DIC, as for phytoelements in small chambers (See The Closed Chamber with Injection Ports), but photosynthetic parameters and their dependence on DIC and temperature are markedly different for communities than phytoelements.

Submerged aquatic plants grow in communities of variable density where the spatial structure and self shading are prominent features (Sand-Jensen, 1989). Light limitation is substantial and the efficiency of photosynthesis at low light is therefore important (Binzer and Sand-Jensen, 2002a,b). The photosynthetic chamber needs to be large enough to include tall communities (Binzer et al., 2006; Middelboe et al., 2006). It is made of glass or transparent acrylic glass and viewed from above, the shape of photosynthetic chambers can be cylindrical, rectangular, or quadratic. The cylindrical shape can be advantageous because the surface area of side walls relative to chamber volume is smaller than in rectangular or quadratic chambers, and these two latter types may also have “dead corners” with stagnant waters. The light sources are high pressure metal halide lamps (mercury or sodium) or light emitting plasma lamps because only those provide a sufficiently high irradiance (>1000 μmol photon m^−2^ s^−1^). The light sources must be placed more than 0.5 m above the photosynthetic chamber and the light path both above the chamber and around the chamber walls are surrounded by totally reflecting material to reduce the influence of distance with depth in the chamber both when plants are absent or present. Irradiance is measured with depth in the water and through canopies of different densities using a small spherical PAR sensor. To ensure statistically reliable determinations of vertical attenuation a series (e.g., 10) of measurements are performed at different positions (Middelboe et al., 2006). Temperature, O_2_, DIC, and pH are set and measured as described in Section “The Closed Chamber with Injection Ports,” while mixing is provided by large submersible pumps ensuring current velocities above 2 cm s^−1^. Temperature control is attained by direct cooling and warming of the water in the incubation chamber or by placing it in a larger temperature controlled holding tank. In the latter case some temperature variations (1–3°C) is difficult to avoid between light and darkness.

Algal communities for measurements can be collected attached to stones or established over a period of one or several years on artificial tiles of desired size set out in the field and later brought to the laboratory for measurements in the photosynthetic chamber (Binzer et al., 2006; Middelboe et al., 2006). Rooted submerged plants can be harvested from natural stands with the 3D structure kept intact when roots and rhizomes are interwoven. In other cases, individual plants are placed in a homogeneous pattern on the chamber bottom in small plastic bags surrounding the root system. Alternatively the individuals are tied to a frame on the chamber bottom. Plant density is determined as fresh mass, dry mass, or plant surface area normalized to bottom area. Leaf area indices (LAI) ranging from 1 to 12 are useful for comparisons among species. Vertical distribution of plant biomass and surface can be determined by cutting the plants sequentially in well defined strata starting at the top of the canopy.

The setup is suited to evaluate the influence on community photosynthesis by variable irradiance, temperature, DIC (including variable CO_2_ and ${\text{HCO}}_{3}^{-}$), canopy density, and spatial structure (Sand-Jensen et al., 2007).

Community photosynthesis can also be determined over longer periods of time by employing the large chambers in an open mode. This allows for exchange of O_2_ and CO_2_ with the atmosphere to prevent that the chambers undergo too extensive accumulation and depletion in the water during several days of alternating light or dark periods. For calculation of photosynthesis and respiration, exchange rates between air and water must be determined. The flux (F_exch_, mol m^−2^ s^−1^) between water and air for O_2_ is given by the equation:

(7) $${\text{F}}_{\text{exch}}\text{ = }K\left({\text{C}}_{\text{act}}-{\text{C}}_{\text{equ}}\right)$$

where *K* is the exchange coefficient (piston velocity, ms^−1^), C_act_ is the actual and C_equ_ is the equilibrium concentration of O_2_ (mol m^−3^) in water at the actual temperature (Staehr et al., 2012b). Piston velocity is controlled by surface turbulence and can, therefore, be considered a constant for a given mixing regime determined by the strength and location of the pumps and the dampening influence of the plant community. Thus, *K* must be directly measured for a given plant density and mixing regime. This is best done in the dark, where only dark respiration (mol m^−2^ s^−1^) takes place, by modifying C_act_ to for example 10 or 30 kPa and measuring the total O_2_ flux (F) over time as a result of respiration and exchange with the atmosphere above from time changes in O_2_ concentrations in the water:

(8) $$\text{F}=\text{R}+\text{K}\left({\text{C}}_{\text{cat}}-{\text{C}}_{\text{aqu}}\right)$$

From 30 kPa the actual pO_2_ will first rapidly decline as a combined result of respiration and loss to the atmosphere and gradually decline less rapidly as pO_2_ approaches equilibrium with the atmosphere and respiration alone drives pO_2_ further downwards. Calculations of pO_2_ changes over time in relation to differences in the pO_2_ gradient between water and air produces a straight line (Eq. 8) permitting calculation of *R* and *K* assuming that they remain constant for a given mixing intensity and plant density.

Community measurements operated in an open mode have the main advantage for future application that fluxes of O_2_, DIC, Ca^2+^, H^+^, and nutrient ions (NH_4_^+^, NO_3_^−,^ and PO_4_^3−^) can be determined during repeated diel light dark cycles for several weeks while the submerged plants may also grow. Combined field measurements have been operated in open chambers and mesocosms under a strict mixing regime under natural temperature and light conditions both for phytoplankton (e.g., Markager and Sand-Jensen, 1989), submerged aquatic plants (e.g., Liboriussen et al., 2005), and flooded terrestrial plants (e.g., Setter et al., 1988).

### The open natural system

Principle: Natural ecosystems dominated by submerged aquatic plants have free undisturbed gas exchange with the atmosphere and input/output of water. Determination of ecosystem metabolism by open water measurements requires accurate calculations of atmospheric exchange of O_2_ and CO_2_. The main advantages of the ecosystem approach is that environmental conditions and processes are natural and temporal patterns can be followed over months or years, while allowing plant density and acclimation to gradients in light, DIC, and other environmental variables to develop.

Photosynthesis of submerged aquatic plants derived from analysis of ecosystems can only be determined when rooted plants or macroalgae are the main phototrophs responsible of more than 90% of ecosystem photosynthesis. Only then can the patterns obtained be referred to the metabolism of macrophytes accepting that a minor error (<10%) due to photosynthesis of microalgae may be present. The dominance of submerged aquatic plants can be realized in shallow plant rich ponds, lakes, streams, and coastal lagoons. Open water measurements are used to follow changes in O_2,_ DIC, pH, temperature, and irradiance, and enable calculation of ecosystem net production, plant gross production, and community respiration assuming fully mixed conditions (Odum, 1956; Staehr et al., 2012a). Meteorological observations of wind direction, wind velocity, atmospheric pressure, etc., in standing waters and current velocity, water depth, slope, and bed roughness in flowing waters, can be used to estimate physical exchange coefficients of gases (i.e. piston velocities) and thus calculate fluxes between water and atmosphere using empirical models (Sand-Jensen and Staehr, 2011). Flow chambers, floating chambers, inert gases, and coverage of water surfaces by impermeable floating plastic can be used for direct determination of exchange coefficients which are critical in all determinations of ecosystem metabolism (Staehr et al., 2012a,b). Rooted plants with gas filled lacunae formation and release of gas bubbles can introduce error. Oxygen storage may delay establishment of steady state exchange of O_2_ following dark light switches by some 10–20 min for most rooted plants (Westlake, 1978) and loss of bubbles is negligible in swift flowing waters, while bubble release may account for 10% of net O_2_ release in slow flowing waters (Kragh et al., unpublished data).

The strength of these measurements is that they provide natural rates under fully realistic and undisturbed environmental conditions. They can reveal the coupling between O_2_ and carbon metabolism, the natural precipitation and dissolution of carbonates and the direct involvement of accumulation and release of acids in the photosynthetic process. Measurements have shown fast exchange rates of protons between macrophytes and water following diurnal light dark switches partly uncoupled from exchanges of DIC during photosynthesis and respiration; a phenomenon that is not unraveled in short term laboratory measurements with detached phytoelements (Kragh et al., unpublished data). Ecosystem measurements can also reveal how early summer growth in biomass and late summer senescence influence plant metabolism and how ongoing desiccation of ponds may suddenly stop photosynthesis and accelerate decomposition, while refilling may restart photosynthesis and growth (Christensen et al., 2013). Modeling approaches, as successfully used for canopy level understanding of terrestrial systems systems (Ainsworth and Long, 2005), should also be applied more widely in studies of aquatic systems (e.g., Binzer and Sand-Jensen, 2002a,b). All techniques for measuring and calculating ecosystem process are basically available (Staehr et al., 2012a) and awaits broad scale application.

## Outlook

Studies of photosynthesis by aquatic and submerged wetland plants are few compared with research on photosynthesis in air, but underwater systems are attracting more attention. Light and CO_2_ availability under water are often low to submerged plants. Low CO_2_ together with impeded escape of O_2_ can result in high photorespiration as a component determining net photosynthesis. Focus studies of contrasting species and systems are required to develop our understanding of “models” since the environment under water is more complex than in air and there is a diversity of photosynthetic mechanisms (i.e. C_3_, C_4_, CAM, and bicarbonate use) in aquatic species.

The physical and chemical environments of overland floods are only poorly known and few data exist on light extinction and CO_2_ and O_2_ concentrations in floodwaters. Such data are crucial to design relevant laboratory experiments on submergence tolerance of terrestrial plants and to establish, for example, carbon budgets during submergence on leaf lamina as well as for whole plants. Also, studies on leaf acclimation of terrestrial plants to facilitate gas exchange and light utilization under water are also only in their infancy; these acclimations influence underwater photosynthesis as well as internal aeration of plant tissues during submergence.

Finally, a challenge also exists to assess the influence of light, inorganic carbon, and temperature on natural aquatic communities of variable density instead of only studying detached leaves in the scenarios of rising CO_2_ and temperature. Use of mathematical modeling, both at the leaf and community levels, will provide valuable additional understanding of underwater photosynthesis. Improved knowledge of plant and environmental factors determining rate of underwater net photosynthesis at various scales (leaf-to-community) is essential for understanding aquatic plant ecophysiology, submergence tolerance of terrestrial plants, and productivity of the many aquatic and flood-prone ecosystems worldwide.

## Conflict of Interest Statement

The authors declare that the research was conducted in the absence of any commercial or financial relationships that could be construed as a potential conflict of interest.
